# RNA *Trans*-Splicing Modulation via Antisense Molecule Interference

**DOI:** 10.3390/ijms19030762

**Published:** 2018-03-07

**Authors:** Bernadette Liemberger, Josefina Piñón Hofbauer, Verena Wally, Claudia Arzt, Stefan Hainzl, Thomas Kocher, Eva M. Murauer, Johann W. Bauer, Julia Reichelt, Ulrich Koller

**Affiliations:** 1 EB House Austria, Research Program for Molecular Therapy of Genodermatoses, Department of Dermatology, University Hospital of the Paracelsus Medical University, 5020 Salzburg, Austria; j.d.pinon@salk.at (J.P.H.); v.wally@salk.at (V.W.); s.hainzl@salk.at (S.H.); t.kocher@salk.at (T.K.); e.murauer@salk.at (E.M.M.); j.reichelt@salk.at (J.R.); 2 Laboratory for Immunological and Molecular Cancer Research, Department of Internal Medicine III with Hematology, Medical Oncology, Hemostaseology, Infectious Diseases, Rheumatology, Oncologic Center, Paracelsus Medical University, 5020 Salzburg, Austria; c.arzt@salk.at; 3 Department of Dermatology, University Hospital of the Paracelsus Medical University, 5020 Salzburg, Austria; jo.bauer@salk.at

**Keywords:** RNA *trans*-splicing, RNA therapy, fluorescence-based screening system, antisense molecules, epidermolysis bullosa, *KRT14*

## Abstract

In recent years, RNA *trans*-splicing has emerged as a suitable RNA editing tool for the specific replacement of mutated gene regions at the pre-mRNA level. Although the technology has been successfully applied for the restoration of protein function in various genetic diseases, a higher *trans*-splicing efficiency is still desired to facilitate its clinical application. Here, we describe a modified, easily applicable, fluorescence-based screening system for the generation and analysis of antisense molecules specifically capable of improving the RNA reprogramming efficiency of a selected *KRT14*-specific RNA *trans*-splicing molecule. Using this screening procedure, we identified several antisense RNAs and short rationally designed oligonucleotides, which are able to increase the *trans*-splicing efficiency. Thus, we assume that besides the RNA *trans*-splicing molecule, short antisense molecules can act as splicing modulators, thereby increasing the *trans*-splicing efficiency to a level that may be sufficient to overcome the effects of certain genetic predispositions, particularly those associated with dominantly inherited diseases.

## 1. Introduction

RNA-based therapies constitute elegant options to restore gene function in various genetic diseases. Therapeutic intervention at the posttranscriptional level during the course of naturally occurring mRNA maturation in the cell represents a viable opportunity to manipulate the mRNA sequence prior to protein translation. Examples of such strategies include splice-modulating therapies, such as antisense oligonucleotide (ASO)-mediated exon skipping and spliceosome-mediated RNA *trans*-splicing (SMaRT), both of which are currently under development for the therapy of several monogenetic diseases [1,2,3].

Others [4,5] and we ourselves [6,7] have applied SMaRT as an emerging RNA editing tool for the specific correction of recessively and dominantly inherited mutations. So far, SMaRT has successfully been implemented in RNA repair studies for genetic disorders such as epidermolysis bullosa (EB) [8,9], muscular dystrophy [4,10], Alzheimer’s disease [11], and Huntington’s disease [12]. SMaRT uses the cell’s own splicing machinery to recombine a rationally designed RNA *trans*-splicing molecule (RTM) with the target pre-mRNA of interest, generating a new chimeric gene product. This is in contrast to group I intron-based (also referred to as ribozyme-based) *trans*-splicing RNAs that act without the help of the endogenous spliceosomal machinery. Group I intron ribozymes are mainly found in protists, bacteria, or bacteriophages [13], and similar to SMaRT they can be used for RNA repair via the replacement of the 3′ part of the mRNA with the 3′ exonic sequence provided by the ribozymes [14,15] or by splicing a suicide gene into the target RNA to kill tumor cells [16,17]. In general, however, the efficiency of both these RNA technologies needs to be significantly improved for a prospective clinical application.

The efficiency and specificity of SMaRT mainly relies on the RTM’s composition and binding affinity to the respective target locus of interest. The RTM carries the wild-type coding region to be inserted into the target pre-mRNA, splicing elements for efficient splicing, and a binding domain (BD) that specifically binds an intron of the target pre-mRNA and is critical for initiating RNA recombination. SMaRT is suitable for the specific exchange of 5′-, 3′-, or internal parts of a given transcript, referred to as 5′, 3′, or double RNA *trans*-splicing [18,19]. Thereby, any pathogenic mutation within the respective gene region can be replaced with a single type of RTM. For EB, a severe skin blistering disease [20], different *trans*-splicing strategies have been described to correct EB-associated mutations in *COL7A1* [7,19], *COL17A1* [9], *KRT14* [6], and *PLEC* [8]. Although important progress in RTM design has been achieved, particularly in the selection of the BD sequence [21,22], the low efficiency of the technology remains an obstacle to its broad in vivo application. However, our first ex vivo and in vivo studies in recessive dystrophic epidermolysis bullosa (RDEB) mouse models showed that a low RNA reprogramming efficiency can be sufficient to restore protein function [7,23]. Via transduction of a *COL7A1*-specific RTM into RDEB keratinocytes using a lentiviral SIN vector, we achieved an RNA repair efficiency of ~2% in an isolated single cell clone, which resulted in restoration of normal levels of type VII collagen [7,24]. As type VII collagen has a reported half-life of two months, at least in murine skin [25], it can be assumed that a low percentage of *COL7A1* repair at the RNA level may be sufficient to restore a wild-type phenotype in DEB (dystrophic EB) skin.

In general, a higher *trans*-splicing efficiency is probably needed for dominant negative mutations which also contribute to the etiology of other EB subtypes, as well as other genodermatoses such as epidermolytic ichthyosis [26,27]. In this regard, Wally et al. demonstrated that RNA *trans*-splicing-mediated repair of a dominant negative *KRT14* mutation in EB simplex (EBS) keratinocytes resulted only in a partial reversion of the phenotype [6]. Thus, a higher reprogramming efficiency is still required for the treatment of dominantly inherited diseases and, certainly for any *trans*-splicing-based in vivo RNA repair strategy, which can be achieved for instance via splicing modulation using short antisense molecules [4,28].

ASOs can be designed to bind mutation-harboring exons during the pre-mRNA splicing process in order to promote their skipping [1,29,30]. In the optimal case, the resulting mRNA, while shorter, is translated into a fully functional protein. The feasibility of this strategy was recently shown in a mouse model, wherein skipping of the mutation-harboring exon 80 resulted in restoration of *COL7A1* function [31]. In this study, however, the outcome of ASO application we explored was not the skipping of a disease-associated exon, but rather the enhancement of *trans*-splicing efficiency due to inhibition of competitive *cis*-splicing events within the target region. Studies in spinal muscular atrophy showed that co-expression of a therapeutic RTM engineered for *SMN2* correction, together with an antisense RNA molecule that specifically blocked the competing *cis*-splice-site, led to an improvement in *trans*-splicing efficiencies both in vitro and in vivo [4,32]. Furthermore, our group recently demonstrated the possibility of enhancing the *trans*-splicing efficiency of a given RTM via antisense RNAs (asRNAs) in the dystrophic subtype of epidermolysis bullosa (DEB) using a GFP-split model system [28].

The aim of this study was to improve the RNA *trans*-splicing efficiency of a previously described therapeutic *KRT14*-RTM [6] by the co-administration of randomly generated asRNAs expressed via a plasmid and rationally designed short ASOs, applied as oligonucleotides, that bind potential splicing elements within the *KRT14* target region. Dominant mutations in *KRT14* are responsible for the generalized severe form of epidermolysis bullosa simplex (EBS-gen sev). Incorporation of the mutant K14 into the intermediate filament (IF) network compromises its integrity, leading to its collapse into protein aggregates under conditions of stress [6,33,34]. Using our established fluorescence-based screening system [21,22] we investigated the impact of *KRT14*-specific antisense molecules on the *trans*-splicing efficiency of our *KRT14*-RTM and on the general *cis*-splicing pattern within the *KRT14* target region. We achieved increased RNA repair levels after the addition of specific antisense molecules of varying lengths in our screening system. Thus, we were able to identify potential splicing modulators that may further improve the RNA *trans*-splicing technology to a level sufficient to support its implementation in a clinical setting.

## 2. Results

### 2.1. Generation of Antisense Molecules for Trans-Splicing Enhancement

**Figure 1:**
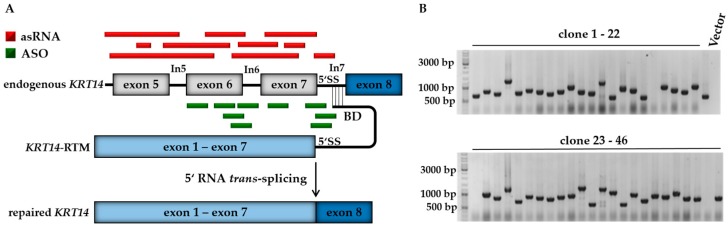
The combined application of antisense strategies. (**A**) The RTM, the main component of the SMaRT system, carries the wild-type coding sequence of *KRT14* spanning from exon 1 to exon 7, splicing elements for efficient splicing and a binding domain specific for intron 7 of *KRT14*. Antisense molecules were randomly (red) and rationally (green) generated in order to block competitive *cis*-splicing elements within the *KRT14* target region, thereby facilitating the *trans*-splicing-induced RNA repair of our pre-selected RTM; (**B**) for asRNA library generation the PCR-amplified *KRT14* target region (1142 bp), spanning from exon 5 to intron 7, was fragmented by sonication and the resulting fragments were randomly cloned into a pcDNA 4.0 expression plasmid. Colony PCR and sequence analysis of individual bacterial clones revealed the presence of various target sequences in sense and antisense orientation. Vector: pcDNA 4.0 expression vector without *KRT14* sequence; RTM, RNA *trans*-splicing molecule; 5′ SS, 5′ splice site.

Aiming to increase the *KRT14* repair efficiency via RNA *trans*-splicing in vitro, we adapted our well established fluorescence-based screening system [21] for the selection of randomly and rationally generated antisense molecules capable of directing the splicing reaction from *cis* to *trans* in the presence of an RTM (Figure 1A). We compared the functionality of asRNAs, some covering the majority of the respective *KRT14* targeting region, and rationally designed ASOs that bind specifically to selected exon–intron boundaries in close proximity to the RTM binding site located within intron 7 of *KRT14* (Figure 1A). Both antisense molecule variants are expected to increase the *trans*-splicing efficiency of the previously described and generated *KRT14*-specific RTM [6] via blocking or interfering with competitive, naturally occurring *cis*-splicing events within the *KRT14* target region. The RTM was designed to replace the first seven exons of *KRT14*, covering all known EBS-associated mutations in this gene. The RTM was previously selected via our fluorescence-based screening system and confirmed to be functional in an EBS patient-derived keratinocyte line [6], although its efficiency warranted improvement to achieve a full phenotypic reversion in these cells.

To generate the asRNA library, the *KRT14* target region encompassing 1144 nt spanning exon 5 to intron 7 immediately 5′ proximal to the BD target site, was PCR-amplified and fragmented via sonication. The resulting fragments were randomly cloned into the pcDNA 4.0 vector (Invitrogen, Carlsbad, CA, USA) downstream to the human cytomegalovirus (CMV) promotor and maintained as individual plasmids expressing *KRT14* sequences in either the sense or antisense orientation with respect to the target region (Figure 1B).

**Figure 2:**
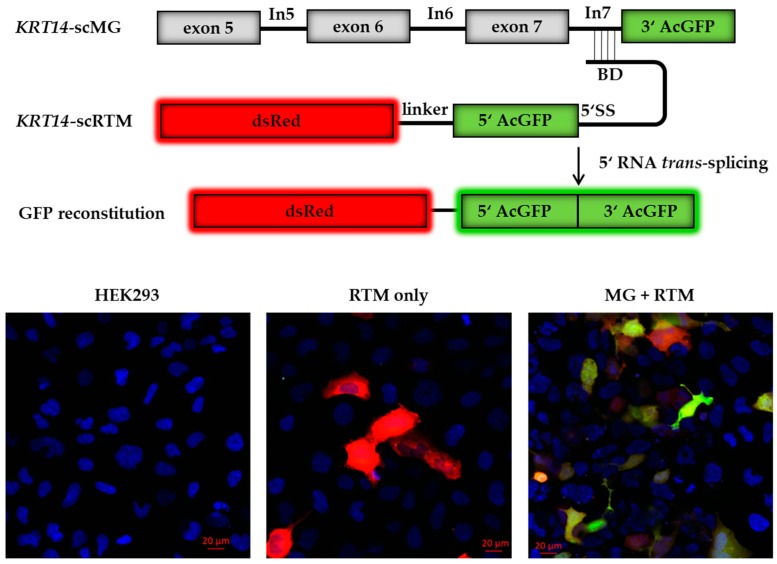
Screening constructs for antisense molecule selection. The reporter-based screening system consists of an artificial *KRT14* screening minigene (*KRT14*-scMG), containing the *KRT14* target region spanning from exon 5 to intron 7 and the 3′ part of GFP, and the RTM screening vector (*KRT14*-scRTM) carrying a dsRed reporter molecule and the missing 5′ GFP part, splicing elements for efficient splicing and a binding domain specific for intron 7 of *KRT14*. Accurate *trans*-splicing between the *KRT14*-scMG and the *KRT14*-scRTM (red) restores the GFP expression (green) in the cell manifested in a yellow signal. Cell nuclei were 4′,6-diamidin-2-phenylindol (DAPI) stained (blue). BD, binding domain; 5′ SS, 5′ splice site.

We obtained a library of 136 asRNAs of varying lengths from 19 nt to 620 nt. Using this library, we initially performed triple transfection experiments, introducing an artificial *KRT14* screening minigene (*KRT14*-scMG; 0.5 µg) and the RTM screening vector (*KRT14*-scRTM; 0.5 µg) together with the individual asRNA plasmids (3 µg). The engineered *KRT14*-scMG consists of the *KRT14* target region from exon 5 to the end of intron 7 (*KRT14* nt: 3320–4462, NCBI Gene ID: 3861) fused to the 3′ half (nt: 337–720) of the GFP coding sequence. The screening RTM is comprised of the dsRed fluorescent reporter molecule sequence, a short linker sequence, the 5′ (nt: 1–337) half of the GFP-coding sequence, a functional 5′ splice site (5′ SS) for efficient splicing, and the BD specific for intron 7 of *KRT14* (Figure 2) [6]. Successful and accurate *trans*-splicing between the pre-mRNA transcribed from the *KRT14*-scMG and the *KRT14*-scRTM resulted in the reconstitution of full-length GFP and translation of a functional protein in treated HEK293 cells detectable by flow cytometry and fluorescence microscopy (Figure 2). The intensity of the GFP signal correlates with the efficiency of the *trans*-splicing reaction. As such, changes in the GFP signal intensity in the presence of the different asRNAs would reflect the positive or negative impact of asRNAs on the *trans*-splicing efficiency [21,22].

**Figure 3:**
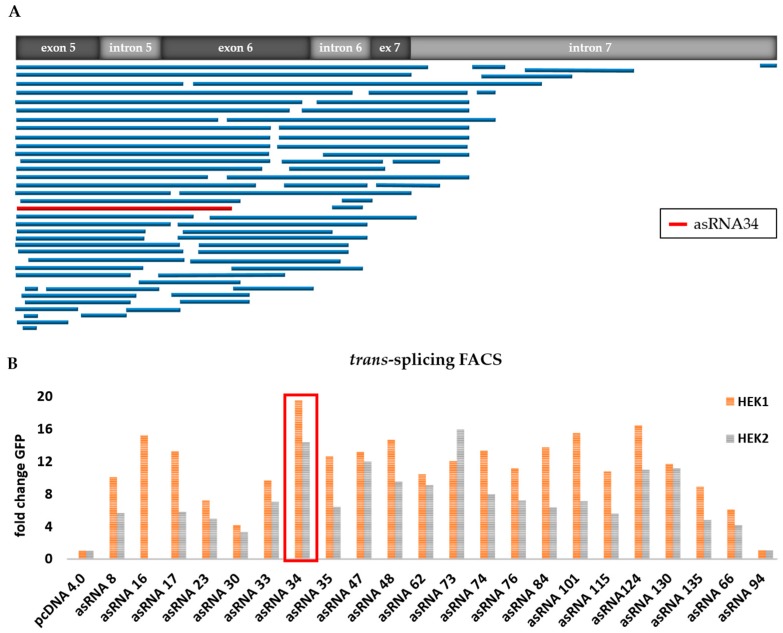
Functional analysis of selected asRNAs. (**A**) Binding position of sequence-analysed individual asRNAs, expressed from the pcDNA 4.0 plasmid, within the *KRT14* target region spanning from exon 5 to intron 7; (**B**) flow cytometric analysis of HEK293 cells upon triple-transfection with the *KRT14*-scRTM, the *KRT14*-scMG and individual asRNAs increased the *trans*-splicing efficiency in comparison to that detectable in HEK293 cells transfected with the screening molecules and an empty (without asRNA sequence) pcDNA4.0 expression plasmid in all transfection experiments. The level of accurate *trans*-splicing was quantified by flow cytometric counting of GFP-expressing cells two days post treatment and referred to the pcDNA4.0 control (set to 1). Two independent transfection experiments (HEK1 and HEK2) are shown.

From the original asRNA library, we initially tested 74 asRNAs (Table 1), varying in length and binding position within the *KRT14* target region (Figure 3A), excluding those that targeted intron 7 in order to avoid direct binding competition between asRNA and RTM to the target pre-mRNA. Flow cytometric analysis revealed an increase in GFP expression levels from three-fold to 20-fold depending on the delivered asRNA (Figure 3B). Based on the initial results, achieved in two rounds of screening (HEK 1 and HEK 2; Figure 3B), we selected asRNA34, one of the most efficient asRNAs, for further investigation.

### 2.2. asRNA34 Facilitates RTM-Mediated RNA Editing

**Figure 4:**
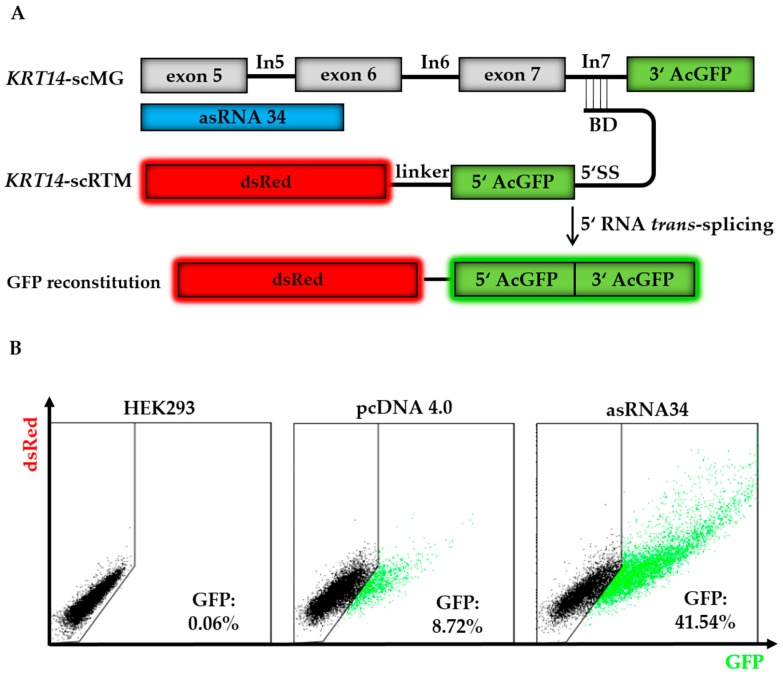
asRNA34 increases *trans*-splicing efficiency in GFP-split model. (**A**) The co-delivery of the screening molecules (*KRT14*-scRTM and *KRT14*-scMG), carrying the respective GFP parts, results in full-length GFP expression upon accurate RNA *trans*-splicing. The addition of asRNA34, specific for *KRT14* exon 5 to exon 6, into HEK293 cells is expected to increase the level of RNA *trans*-splicing represented by the amount of restored GFP in the cells; (**B**) triple-transfection experiments with the *KRT14-sc*MG, the *KRT14*-scRTM, and asRNA34 in HEK293 cells led to an increase in GFP-expressing cells from ~9% (empty pcDNA4.0) to ~42%, measured by flow cytometry. BD, binding domain; 5′ SS, 5′ splice site.

We performed further triple transfection experiments in the HEK293 system using asRNA34 in order to confirm the results from our library screen (Figure 4A). In all described experiments, the total amount of DNA transfected was the same in each setting to exclude variabilities that could arise from transfection efficiency and cell toxicity when using different amounts of DNA. We used pcDNA 4.0 empty vector where needed to ensure equal levels of transfected plasmids. Combining the results of all experiments, we were able to achieve full-length GFP expression in ~42% of HEK293 cells treated with the *KRT14*-scMG (0.5 µg), *KRT14*-scRTM (0.5 µg), and asRNA34 (3 µg). In contrast, in cells receiving pcDNA4.0 (3 µg) instead of asRNA34, only ~9% of treated cells were GFP-positive by flow cytometric analysis (Figure 4B). The geometric mean of GFP-positive cells was 3.7 in the absence of asRNA34, and rose to 13.2 in the presence of asRNA34, representing a 3.6-fold increase in GFP intensity.

**Figure 5:**
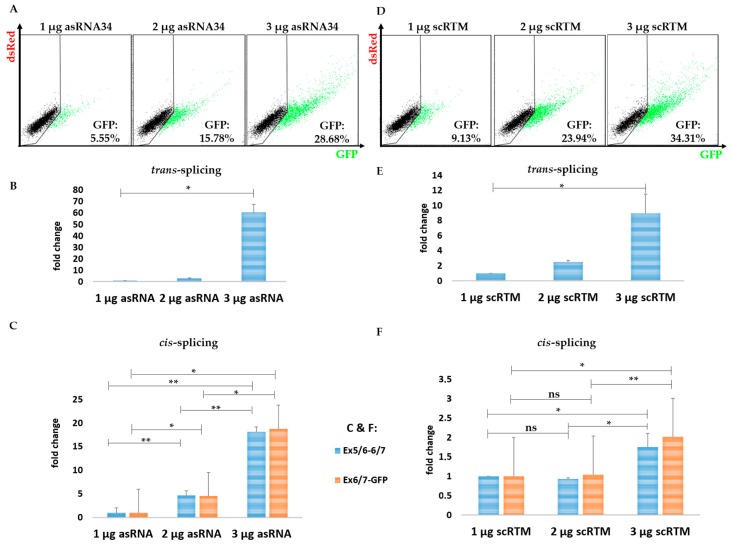
The impact of asRNA34 on *cis*-splicing in the fluorescence-based setting. The transfection experiments further revealed an asRNA34 dose-dependent increase of the *trans*-splicing efficiency confirmed via flow cytometric analysis at protein level (**A**) and via sqRT-PCR at mRNA level (**B**). The asRNA34-mediated *trans*-splicing increment was accompanied by increased *cis*-splicing levels, detected via sqRT-PCR and normalized to GAPDH mRNA levels (**C**) (the mean value + SD of one representative experiment is shown), indicating a stabilizing effect on the target pre-mRNA by antisense molecule interference. Empty pcDNA 4.0 expression plasmids were transfected into HEK293 cells to adjust total plasmid levels delivered into the target cells. Increasing the amount of transfected RTMs results in an increased GFP expression as detected via flow cytometry (**D**) and sqRT-PCR (**E**)**,** although the *cis*-splicing rate within the *KRT14*-scMG remains largely unaffected (**F**). An unpaired Student’s *t*-test (two-tailed) was performed with the GraphPad Prism software (GraphPad Software, San Diego, CA, USA) to prove statistical significance (Table 2 and Table 3). Significance values are given in asterisks (ns = not significant; * *p*-value ≤ 0.05; ** *p*-value ≤ 0.01).

Additionally, asRNA34 had a dose-dependent effect on GFP expression on the protein level, as assayed by flow cytometry and on RNA expression, evaluated by semi-quantitative real time PCR (sqRT-PCR) (Figure 5A,B). Together, these results revealed an increase in *trans*-splicing efficiency of up to 60-fold in the presence of 3 µg asRNA34 instead of 1 µg (Figure 5B). asRNA34 has a length of 324 nt and is predicted to be complementary to a region of *KRT14* spanning the exon 5–intron 5 and intron 5–exon 6 junctions (Figure 4A). As such, asRNA34 is expected to impact *cis*-splicing of intron 5 and not intron 7. To understand how interfering with *cis*-splicing of intron 5 could positively impact *trans*-splicing, we first investigated the impact of increasing asRNA34 concentrations on *cis*-splicing levels at all exon–exon junctions within the *KRT14*-scMG pre-mRNA in the experiments described above. Towards this end, primers spanning the exon–exon junctions were designed to assess the number of mRNA molecules harbouring correctly spliced exons by sqRT-PCR. The analysis of competitive *cis*-splicing events within the *KRT14*-scMG upon co-delivery of different levels of asRNA34-expressing plasmids indicated a potential stabilizing effect of the asRNA on the target pre-mRNA as increased *cis*-splicing levels were detectable via sqRT-PCR analysis (Figure 5C). This finding was contrary to our initial assumption that blocking potential splicing elements within the *KRT14*-scMG resulted in reduced *cis*-splicing rates. In contrast, the amount of transfected RTM primarily impacted only the *trans*-splicing level, and not the *cis*-splicing level as shown via flow cytometric analysis (Figure 5D) and sqRT-PCR (Figure 5E,F).

### 2.3. Splice Site-Specific Antisense Oligonucleotides Increase RNA Trans-Splicing Levels

**Figure 6:**
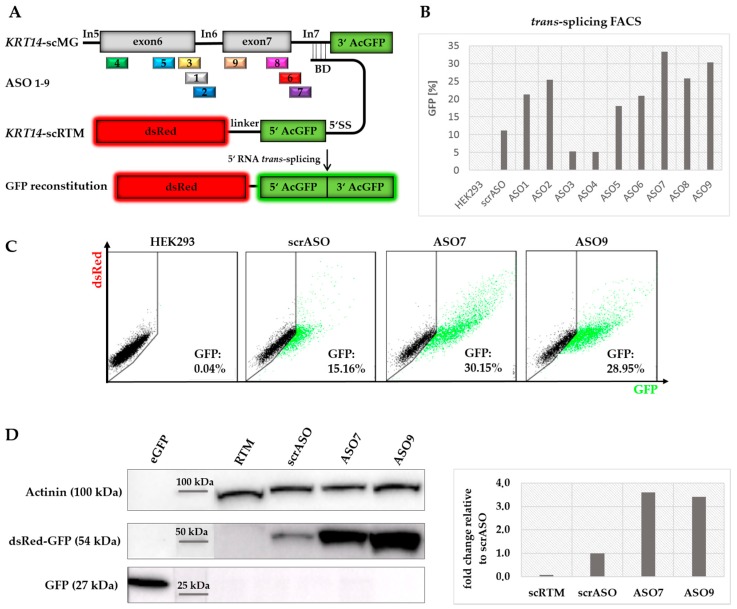
Comparison of rationally designed ASOs. (**A**) ASOs were designed specifically for the exon 6 to intron 7 junctions within the *KRT14* target region upstream to the RTM binding site; (**B**) in contrast to the scrASOs, acting as mock control in the experiment, seven ASOs were capable of increasing the *trans*-splicing activity of the RTM at an ASO concentration of 70 nmol, quantified via flow cytometric analysis; (**C**) the most promising ASOs (ASO7 and ASO9), specific for exon 7 and the exon 7–intron 7 junction of *KRT14*, induced the *trans*-splicing-mediated GFP restoration in 30% (ASO7) and 29% (ASO9) of all analysed cells, whereas in the mock control (scrASO) only 15% of the cell population expressed GFP; (**D**) Western blot analysis revealed an over three-fold increase of full-length dsRed-GFP (54 kDa) expression at protein level in HEK293 cells treated with ASO7 and ASO9 in comparison to cells treated with the scrASO. eGFP (27 kDa) was used as positive control to confirm the specificity of the antibody. The loading control α-actinin (100 kDa) was used for normalization.

Although asRNA34 could confer a significant improvement on the *KRT14*-scRTM’s RNA *trans*-splicing efficiency, we also observed increased *cis*-splicing of intron 7 with increasing concentrations of the asRNA, which would be in direct competition with our desired *trans*-splicing reaction. To avoid the unwanted effect on *cis*-splicing, we generated shorter site-specific ASOs (Table 4) designed to directly block competitive 5′ splice sites (ASO1, 2, 3, 6, 7, 8), potential splice site enhancer sequences (ASO4, 9) (Figure S1), or both, within the *KRT14*-scMG sequence. In total, nine ASOs 20–24 nts in length were rationally designed to cover exon 6 to intron 7 including the exon–intron junctions upstream of the RTM binding site within the *KRT14* target region (Figure 6A). Coady et al. (2008) recently showed that the inhibition of *cis*-splicing of adjacent introns can have a positive impact on the *trans*-splicing efficiency of a given RTM [32]. Therefore, in addition to ASOs specific for the exon–intron 7 junction, we also tested ASOs 1, 2, and 3, which target the exon–intron 6 junction further upstream to the RTM binding site. A scrambled ASO (scrASO), which was designed not to target any region within the *KRT14*-scMG, was included as a control in initial triple transfection experiments. Flow cytometric analysis of transfected HEK293 cells revealed a significant increase of GFP-expressing cells from 11% (scrASO) up to 33% depending on the ASO introduced (Figure 6B). ASO7 and ASO9, binding the exon 7–intron 7 junction and the 5′ end of exon 7, respectively, represented the most promising ASOs, resulting in 33% and 30% GFP-positive cells in the screen. We confirmed the results with these two ASOs in additional experiments, obtaining a similar increase in GFP-positive cell numbers from 15% (scrASO) to 30% (ASO7) and 29% (ASO9) (Figure 6C). Furthermore, Western blot analysis of whole cell lysates from triple-transfected HEK293 cells validated the elevation of *trans*-spliced chimeric products as indicated by an increase in GFP expression (Figure 6D).

**Figure 7:**
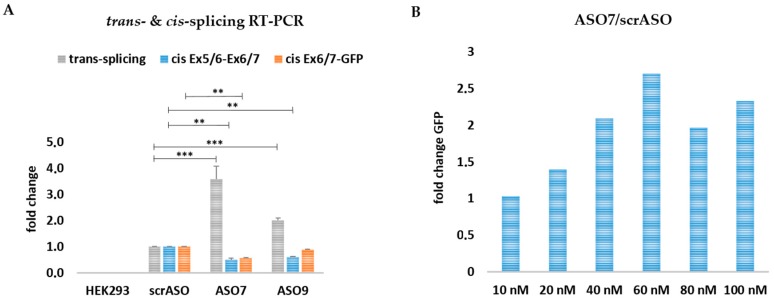
Comparison of rationally designed ASOs on RNA level. (**A**) sqRT-PCR analysis revealed a ~4-fold increase in GFP expression in ASO7 and a ~2-fold increase in ASO9-treated cells in comparison to cells treated with the scrambled ASO. An unpaired Student’s *t*-test (two-tailed) was performed with GraphPad Prism software (GraphPad Software, San Diego, CA, USA) to prove the statistical significance between scrASO and ASO7, as well as scrASO and ASO9 (Table 5). The *cis*-splicing activity within the Ex5/6-Ex6/7 *KRT14*-scMG region was significantly decreased for ASO7 and ASO9. Within the Ex6/7-GFP region the *cis*-splicing activity was decreased for ASO7 but unaffected for ASO9; (**B**) the co-delivery of the *KRT14*-scMG, the *KRT14*-scRTM, and different ASO7 concentrations into HEK232 cells resulted in the most promising *trans*-splicing efficiency at an ASO concentration of 60 nmol. HEK293 cells treated with the screening constructs and respective scrambled ASO concentrations were set to 1. Significance values are given in asterisks (** *p*-value ≤ 0.01; *** *p*-value ≤ 0.001).

We additionally verified this increase in *trans*-splicing efficiency at the mRNA level via sqRT-PCR analysis (Figure S2), revealing an up to 2–4-fold increase in the levels of reconstituted GFP mRNA in cells harboring the selected ASOs as compared to scrASO-treated controls (Figure 7A). In contrast to the results obtained with asRNA34, the shorter ASO7 and ASO9 had no significant effect on the general *cis*-splicing events occurring within the *KRT14*-scMG. Rather the levels of *cis*-splicing within the target pre-mRNA were slightly reduced (~2-fold) in the presence of ASO7, whereas ASO9 exhibited no significant impact on these events (Figure 7A). Increasing the concentrations of ASO7 added to the *trans*-splicing reaction, revealing the highest *trans*-splicing efficiency at a concentration of 60 nmol (Figure 7B).

## 3. Discussion

In recent years, RNA *trans*-splicing has been predominantly applied in RNA repair studies for various genetic diseases or for the RTM-mediated delivery of “suicide genes” into tumour cells [35,36]. Reprogramming at the pre-mRNA level represents an elegant approach for the editing of aberrant RNA molecules that maintains endogenous regulation of the target transcript, making the technology potentially safe and well suited for the therapy of certain genetic conditions. These include disorders caused by mutations affecting large genes or dominantly inherited mutations, difficult to correct via conventional full-length cDNA replacement strategies. In this respect, we have already demonstrated promising results with a *COL7A1*-targeting RTM capable of correcting ~40% of all RDEB-associated mutations encoded within the ~3300-nucleotide long target pre-mRNA [7]. In the case of dominant negative mutations in EBS-gen sev, Cao et al. showed with an inducible EBS mtK14 mouse model, that dominance of the mutation is dependent on the ratio of wild-type to mutated alleles. A reduction in expression of the mutated allele by ~50% appeared sufficient to overcome the EBS phenotype [37]. In this respect, RNA *trans*-splicing-mediated correction of the mutant RNA is expected to not only increase the amount of wild-type *KRT14* transcripts, but also simultaneously decrease the level of mutant transcripts by competing with the normal *cis*-splicing events. Indeed, we could achieve partial reversion of the phenotype of EBS keratinocytes associated with a dominant-negative mutation in *KRT14* with an appropriate *KRT14*-targeting RTM [6].

With the recent advances in the identification and selection of the BD for the RTM [18,21,22], the main obstacle that remains is the low efficiency of the technique, which limits its potential for future in vivo or clinical application. Therefore, we have developed a fluorescence-based screening system aimed at identifying factors with the potential to increase the *trans*-splicing efficiency, such as antisense RNA molecules [28]. In the course of this study, we could identify antisense RNA molecules capable of enhancing the *trans*-splicing efficiency induced by the *KRT14*-targeting RTM by up to 20-fold. The system proved practical for the screening of an unbiased library of randomly generated sequences of varying lengths and binding positions along the target pre-mRNA, as well as short, site-specific ASOs rationally designed to block splice sites and splicing enhancer elements. Our results revealed both antisense strategies to be attractive for future RNA repair studies in patient cells, but also highlight several points for consideration.

In the described screening system, the *KRT14*-scRTM induced a *trans*-splicing reaction between the 5′ GFP coding sequence on the RTM and the 3′ GFP sequence located immediately after intron 7 on the target pre-mRNA molecule. Notably, some of the best-acting antisense molecules identified (asRNA34 and ASO9) were not expected to directly inhibit the competing *cis*-splicing of intron 7. ASO9 was expected to bind to the 5′ region of exon 7, whereas asRNA34 bound a 324-nt region spanning all of exon 5, intron 5, and half of exon 6. As such, asRNA34 was rather expected to interfere with *cis*-splicing of intron 5, whereas ASO9 may or may not interfere with *cis*-splicing of intron 6. The mechanisms by which these molecules enhanced *trans*-splicing efficiency are not yet completely understood. However, blocking potential splicing enhancer and silencer sequences located within the *KRT14*-scMG is likely to impact both types of splicing reactions (*cis* and *trans*). asRNA34, in particular, would block many predicted enhancer and silencer sequences, and thereby affect the general spliceosome-mediated splicing efficiency (Figure S3) in an unpredictable manner. In this respect, increasing amounts of asRNA34 added to the cells led to a significant enhancement of both *cis*- and *trans*-splicing reactions. Besides blocking potential splicing motifs within the MG sequence, asRNA34 may also exert a general stabilizing effect on the target pre-mRNA that promotes both *cis*- and *trans*-splicing reactions. Thus, while we achieved increased *trans*-splicing rates by the addition of asRNA34, the concurrent upregulation of *cis*-splicing in the target pre-mRNA would be expected to negate any of its associated benefits, as no alteration in the ratios of wild-type (corrected) transcripts to mutant transcripts can be expected.

In contrast to long asRNA sequences, the mechanisms of action by which short ASOs exert their effects are more clearly defined, as they are designed to inhibit splicing either by directly blocking the splice donor/acceptor sites located within the exon–intron junctions (as in the case of ASO7), or by blocking potential splicing enhancer motifs adjacent to the intron–exon junction (ASO9). Both ASO7 and ASO9 resulted in enhanced *trans*-splicing of the RTM without any detectable increase in *cis*-splicing events within the target-pre-mRNA, suggesting that these shorter ASOs are better suited for our purposes. The most auspicious ASO7, targeting the exon 7–intron 7 boundary, additionally exhibited an inhibitory effect on the competitive *cis*-splicing of intron 7 as expected. The observed 2-fold inhibition in *cis*-splicing of intron 7, combined with the 4-fold increase in *trans*-splicing will not only reduce the level of mutated transcripts in the patient cells but also shift the ratio of wild-type to mutant transcripts further towards wild-type. This is expected to decrease the dominant negative effect of the disease-causing mutation to a level that may be sufficient to fully restore the wild-type phenotype in patient cells.

This study describes a straightforward and practical protocol that can be adapted to the screening of any type of splicing modulator, including pharmacological compounds. Our results also demonstrate that there are different mechanisms by which antisense technologies can impact splicing events and further underscore the need to comprehensively evaluate the effects of such molecules on different processes and outcomes including, for example, RNA stability. To this end, future studies will not only focus on evaluation of this therapeutic strategy in EBS patient-derived keratinocytes, but also on the investigation of mechanisms of action and precise quantification of corrected and mutant transcripts using next-generation sequencing platforms.

## 4. Materials and Methods

### 4.1. Construction of asRNA Library and Antisense Oligonucleotides

An asRNA library was constructed by the fragmentation of the PCR-amplified *KRT14* target region spanning from exon 5 to intron 7 by sonication according to an already established protocol [21,28]. PCR amplification was performed using genomic DNA from a healthy donor and the GoTaq DNA polymerase (Promega, Madison, MI, USA). The resulting fragments were treated with a DNA Terminator End Repair Kit (Lucigen, Middleton, WI, USA), cloned into a pcDNA 4.0 plasmid (Invitrogen, Carlsbad, CA, USA) using the restriction site for EcoRV, and analyzed for their correct orientation (complementary to the target region) by sequence analysis. PCR products were digested with the corresponding restriction enzymes for 1 h at 37 °C, purified with a Illustra GFX PCR DNA and Gel Band Purification Kit (GE Healthcare, Chalfont St. Giles, UK) and ligated with T4 DNA ligase (Thermo Fisher Scientific, Waltham, MA, USA), all according to the manufacturer’s protocols. The ligation was then transformed into chemically competent SoloPack^®^ Gold Competent Cells (Agilent Technologies, Santa Clara, CA, USA) and plasmid preparations were carried out using a Plasmid Mini Prep Kit (Sigma-Aldrich, Taufkirchen, Germany), according to the manufacturer’s protocol. Sequence analysis of all plasmids and PCR products was performed using a 3500 ABI automated sequence analyzer and ABI PRISM dye terminator cycle sequencing kit (Applied Biosystems, Foster City, CA, USA).

### 4.2. Screening Constructs

Screening constructs used for detection of *cis*-splicing and correct *trans*-splicing were designed and cloned according to the protocol of Bauer et al. [21]. Concisely said, the *KRT14*-scRTM carries a 5′ split-GFP part consisting of the first nucleotides (nt: 1–336) from a full length acGFP (pacGFP vector, Clontech, Mountain View, CA, USA) and a BD sequence that specifically targets Intron 7 of *KRT14* cloned into the pcDNA3.1D/V5-HIS (Invitrogen, Carlsbad, CA, USA) backbone. The *KRT14*-scMG spanning exon 5 to intron 7 was generated using a *KRT14* exon 5 forward primer (5′-GATCAAGCTTCACCACAGAGGAGCTGAACCGCGAGGTGGC-3′) and a *KRT14* intron 7 reverse primer (5′-GATCGGATCCGGGGAAGAGGTGGGAAGAGGACGTTACC-3′) for amplification via GoTaq DNA polymerase (Promega, Madison, MI, USA). Afterwards, the amplified PCR product was cloned into the screening vector backbone downstream to the CMV-promoter (pcDNA3.1, Invitrogen, Carlsbad, CA, USA) using HindIII and BamHI restriction sites [21,28]. The *KRT14*-scMG also carries the 3′ split-GFP portion consisting of the rearward part of acGFP (nt: 337–720) to enable a reconstitution of the full length acGFP upon accurate *trans*-splicing.

### 4.3. Cell Culture and Plasmid Transfection

For triple-transfection studies, the human embryonic kidney cell line HEK293 (Stratagene, La Jolla, CA, USA) was used and grown in Dulbecco’s Modified Eagle’s Medium (DMEM) supplemented with 10% fetal bovine serum (FBS) and 100 U/mL penicillin–streptomycin (Biochrom, Berlin, Germany) at 37 °C and 5% CO_2_ in a humidified incubator. Passaging of the cells was carried out with Trypsin (0.05%)-EDTA (0.02%) (Biochrom, Berlin, Germany) treatment followed by Trypsin-EDTA inactivation with Solution A (7.25 g HEPES; 1.8 g glucose; 0.22 g NaCl; 0.27 g Na_2_HPO_4_; 1 mL phenol red; 900 mL H_2_O; pH 7.4) supplemented with 10% FBS. Cells were centrifuged at 350× *g* for 5 min and seeded in fresh tissue culture 6-well plates or 60 mm plates.

Transient transfections of screening plasmids were performed the next day using the jetPEI reagent (Polyplus-transfection SA, Illkirch, France) according to the manufacturer’s protocol. The transfected DNA amounts varied in the different experiments: Section 2.2: 0.5 µg *KRT14*-MG + 0.5 µg RTM + 3 µg asRNA were transfected into HEK293 cells cultivated in 6-well plates; Section 2.3: 0.5 µg *KRT14*-MG + 0.5 µg RTM + 70 nM ASO were transfected into HEK293 cells cultivated in 6-well plates.

### 4.4. Flow Cytometric Analysis

The amount of *trans*-splicing events, manifested in the expression of GFP, was measured 48–72 h after transfection using a Beckman Coulter FC-500 FACS analyzer (Beckman Coulter, Vienna, Austria). The transfected HEK293 cells were washed once with phosphate buffer saline (Dulbecco’s PBS) and detached from the cell culture plates using Trypsin-EDTA. Trypsin-EDTA was inactivated with Solution A (7.25 g HEPES; 1.8 g glucose; 0.22 g NaCl; 0.27 g NaHPO_4_; 1 mL phenol red; 900 mL H_2_O; pH 7.4) supplemented with 10% FBS. After centrifugation for 5 min at 350× *g*, 10,000 cells were analyzed according to their GFP expression and geometric mean with the Kaluza 1.3 software (Beckman Coulter, Brea, CA, USA).

### 4.5. RNA Isolation and cDNA Synthesis

HEK293 cells were harvested 48–72 h post transfection and RNA was isolated using the Innuprep RNA Mini Kit (Analytik Jena, Jena, Germany) according to the manufacturer’s protocol. Afterwards cDNA synthesis was performed with 3 µg RNA using the iScript™ cDNA Synthesis Kit (BioRad, Hercules, CA, USA) according to the manufacturer’s protocol.

### 4.6. sqRT-PCR Analysis of Cis- and Trans-Splicing Levels

sqRT-PCR was performed to detect fused GFP transcripts in treated HEK293 cells. For sqRT-PCR analysis, a 5′ GFP-specific forward primer (5′-GCTGACCCTGAAGTTCATCTG-3′), a 3′ GFP-specific reverse primer (5′-CGCCGATGGGGGTATTCTGCTGG-3′), cDNA of treated cells and GoTaq^®^ qPCR Master Mix (Promega, Madison, WI, USA) was used. The PCR was performed using a Bio-Rad CFX™ system (BioRad, Hercules, CA, USA) with the following conditions: 95 °C for 2 min, and 50 cycles of 20 s at 95 °C, 20 s at 62 °C, and 25 s at 72 °C. Experiments were carried out in duplicates and repeated two times. Correct PCR products were verified by direct sequence analysis.

To analyze differences in *cis*-splicing events after transfection of HEK293 cells exon–exon junction-specific forward (Exon5/6: 5′-AGCTCAGCATGAAAGCATCCCT-3′, Exon6/7: 5′-AGGACGCCCACCTCTCCTCC-3′) and reverse (Exon6/7 5′-GGAGGAGAGGTGGGCGTCCT-3′, GFP: 5′-GGTCAGCTCGATGCGATTCACC-3′) primers, cDNA of treated cells and GoTaq^®^ qPCR Master Mix (Promega, Madison, WI, USA) was used. The PCR was performed using a Bio-Rad CFX™ system with the following conditions: 95 °C for 2 min, and 50 cycles of 20 s at 95 °C, 20 s at 64 °C, and 25 s at 72 °C. Experiments were carried out in duplicates and repeated two times. Correct PCR products were verified by direct sequence analysis.

### 4.7. Western Blot Analysis

Enhancement of asRNA/ASO-induced *trans*-splicing manifests in an increase in full-length GFP expression, which was quantified at the protein level via Western blot analysis 48–96 h post transfection. HEK293 cells were washed with PBS, harvested, and resuspended in radioimmunoprecipitation assay buffer RIPA (Santa Cruz Biotechnology, Heidelberg, Germany). Samples were denatured for 5 min at 95 °C in 4× SDS loading buffer (0.25 M Tris-HCl; 8% SDS; 30% glycerol; 0.02% bromphenol blue; 0.3 M β-mercaptoethanol; pH 6.8). SDS-gel-electrophoresis was performed at 140 V for a maximum of 2 h using a NuPAGE 12% Bis-Tris gel (Invitrogen, Carlsbad, CA, USA) and NuPAGE MOPS running buffer. Proteins were electro-blotted onto a nitrocellulose membrane (Amersham Hybond-ECL, Amersham, Buckinghamshire, UK) at 0.25 A for 75 min. The membrane was blocked with Western Blocking Reagent (Roche, Basel, Switzerland) in Tris buffered saline (TBS) + 0.2% Tween (TBS-T) for 1 h at RT and incubated with a primary anti-GFP rabbit IgG antibody (MBL-598, MBL International, Woburn, MA, USA) in a dilution of 1:750 in TBS-T at 6 °C overnight. After three washing steps (3 × 10 min) with TBS-T the membrane was incubated with a secondary HRP Envision+ labelled anti rabbit antibody (1:100 in TBS-T, Agilent Technologies, Santa Clara, CA, USA) and incubated for 90 min at room temperature. Finally, GFP expression was visualized using the Luminata™ Forte Western HRP Substrate (Millipore, Burlington, MA, USA). α-actinin (H-2) mouse monoclonal IgG antibody (1:1000 in TBS-T; Santa Cruz. Dallas, TX, USA) and a secondary HRP Envision+ labelled anti mouse antibody (1:100 in TBS-T Tween, Agilent Technologies, Santa Clara, CA, USA) were used to detect α-actinin which served as protein loading control. The relative quantification of GFP expression was measured and calculated using the Image Lab 5.2.1 (Bio-Rad) software.

### 4.8. Statistical Analysis of SqRT-PCR Data

For statistical analysis, an unpaired Student’s *t*-test (two-tailed) was performed with the GraphPad Prism 5.03 software (GraphPad Software, San Diego, CA, USA) to prove statistical significance between the values of controls (pcDNA 4.0 & scrASO) and samples (asRNA34, ASO7, ASO9). A sample size of at least *n* = 4 was analyzed for mean ± SEM to provide a *p*-value.

### 4.9. Analysis of Splicing Enhancer and Silencer

DNA sequence motifs, that probably regulate/influence the general splicing mechanism within the *KRT14* targeting region, were predicted using the prediction websites http://regrna.mbc.nctu.edu.tw/html/prediction.html and http://www.umd.be/HSF3/HSF.shtml [38].

## Figures and Tables

**Table 1:** Randomly generated asRNAs.

	Length (nt)	*KRT14* Binding Position (nt)					Length (nt)	*KRT14* Binding Position (nt)			
		from		to				from		to	
asRNA1	42	477	In6	518	In6	asRNA68	174	9	Ex5	182	In5
asRNA2	110	246	Ex6	355	Ex6	asRNA69	20	13	Ex5	32	Ex5
asRNA3	117	235	Ex6	351	Ex6	asRNA70	20	13	Ex5	32	Ex5
asRNA4	148	183	In5	330	Ex6	asRNA71	380	1	Ex5	380	Ex6
asRNA5	150	409	Ex6	558	Ex7	asRNA72	193	1	Ex5	193	In5
asRNA7	155	529	In6	683	In7	asRNA73_1	74	563	Ex7	636	In7
asRNA8	163	12	Ex5	174	In5	asRNA73_2	21	1122	In7	1142	In7
asRNA13	192	216	In5	407	Ex6	asRNA76_1	27	693	In7	719	In7
asRNA14	203	324	Ex6	526	In6	asRNA76_2	246	1	Ex5	246	Ex6
asRNA15	220	464	In6	683	In7	asRNA78	118	327	Ex6	444	In6
asRNA16	225	264	Ex6	488	In6	asRNA81	217	1	Ex5	217	In5
asRNA17	228	275	Ex6	502	In6	asRNA83	592	1	Ex5	592	In7
asRNA18	228	275	Ex6	502	In6	asRNA84	230	1	Ex5	230	Ex6
asRNA19	228	254	Ex6	481	In6	asRNA86	308	292	Ex6	599	In7
asRNA20	245	6	Ex5	250	Ex6	asRNA87	287	1	Ex5	287	Ex6
asRNA21	230	452	In6	681	In7	asRNA92_1	119	1	Ex5	119	Ex5
asRNA22	255	429	Ex6	683	In7	asRNA92_2	178	1	Ex5	178	In5
asRNA23	243	18	Ex5	260	Ex6	asRNA94	96	1	Ex5	96	Ex5
asRNA24	289	395	Ex6	683	In7	asRNA97	95	541	Ex7	635	In7
asRNA25	288	394	Ex6	681	In7	asRNA99	119	1	Ex5	119	Ex5
asRNA26	291	392	Ex6	682	In7	asRNA100	127	401	Ex6	527	In6
asRNA29	366	318	Ex6	683	In7	asRNA101	195	2	Ex5	196	In5
asRNA30	377	8	Ex5	384	Ex6	asRNA105	251	1	Ex5	251	Ex6
asRNA31	379	1	Ex5	379	Ex6	asRNA109	435	1	Ex5	435	Ex6
asRNA32	406	316	Ex6	721	In7	asRNA111	164	766	In7	929	In7
asRNA33	386	1	Ex5	386	Ex6	asRNA113	192	1	Ex5	192	In5
asRNA34	324	1	Ex5	324	Ex6	asRNA115	506	1	Ex5	506	In6
asRNA35	414	1	Ex5	414	Ex6	asRNA116	362	1	Ex5	362	Ex6
asRNA39	303	1	Ex5	303	Ex6	asRNA119	19	15	Ex5	33	Ex5
asRNA47	235	1	Ex5	235	Ex6	asRNA120	44	492	In6	535	In6
asRNA48	168	46	Ex5	213	In5	asRNA124	85	167	In5	251	Ex6
asRNA53	382	1	Ex5	382	Ex6	asRNA125	272	1	Ex5	272	Ex6
asRNA59	287	244	Ex6	530	In6	asRNA130	330	8	Ex6	337	Ex6
asRNA60	620	1	Ex5	620	In7	asRNA131	132	700	In7	831	In7
asRNA62	287	244	Ex6	530	In6	asRNA133	68	96	Ex5	163	In5
asRNA64	346	242	Ex6	587	In7	asRNA134	150	401	Ex6	550	Ex7
asRNA66	519	266	Ex6	784	In7	asRNA135	368	1	Ex5	368	Ex6

Additionally, 20–24 nt antisense oligonucleotides were rationally designed to target the region from exon 6 to intron 7 and ordered with phosphorothioate (PS)-linkage modifications from Microsynth (Microsynth AG, Balgach, Switzerland).

**Table 2:** Statistical significance *trans*-splicing Figure 5B,E.

*Trans*-Splicing	*p*-Value	Significance
1 µg asRNA34 vs. 2 µg asRNA34	0.0492	*
1 µg asRNA34 vs. 3 µg asRNA34	0.0229	*
2 µg asRNA34 vs. 3 µg asRNA34	0.0244	*
1 µg scRTM vs. 2 µg scRTM	0.0419	*
1 µg scRTM vs. 3 µg scRTM	0.0021	*
2 µg scRTM vs. 3 µg scRTM	0.0325	*

Significance values are given in asterisks (* *p*-value ≤ 0.05).

**Table 3:** Statistical significance *cis*-splicing Figure 5C,F.

*Cis*-Splicing	*p*-Value	Significance
*cis*-splicing exon 5/6-6/7		
1 µg asRNA34 vs. 2 µg asRNA34	0.0095	**
1 µg asRNA34 vs. 3 µg asRNA34	0.003	**
2 µg asRNA34 vs. 3 µg asRNA34	0.0056	**
1 µg scRTM vs. 2 µg scRTM	0.6587	ns
1 µg scRTM vs. 3 µg scRTM	0.0291	*
2 µg scRTM vs. 3 µg scRTM	0.0155	*
*cis*-splicing exon 6/7-GFP		
1 µg asRNA34 vs. 2 µg asRNA34	0.0459	*
1 µg asRNA34 vs. 3 µg asRNA34	0.0247	*
2 µg asRNA34 vs. 3 µg asRNA34	0.0401	*
1 µg scRTM vs. 2 µg scRTM	0.8726	ns
1 µg scRTM vs. 3 µg scRTM	0.0175	*
2 µg scRTM vs. 3 µg scRTM	0.0018	**

Significance values are given in asterisks (ns = not significant; * *p*-value ≤ 0.05; ** *p*-value ≤ 0.01).

**Table 4:** Rationally designed ASOs.

ASO	Sequence	nt
ASO1	GCCAAGACTCACTGGGCGTC	20
ASO2	GGAGGGCCAAGACTCACTGG	20
ASO3	CTCACTGGGCGTCCTCGCCC	20
ASO4	GCTGCATGCAGTAGCGACCTTTGG	24
ASO5	TCTCCTGCTCCAGCCGCGTC	20
ASO6	GAGGGTCTTACCATCTCTGG	20
ASO7	CTGCAGAGGAGGAGGGTCTTACC	23
ASO8	CATCTCTGGATGACTGCGAT	20
ASO9	CCAGAGGAGAACTGGGAGGAGG	22
scrASO2	TGTGGCGAGTAGACTCGAAG	20

**Table 5:** Statistical significance *cis*-splicing Figure 7.

*Trans*- and *Cis*-Splicing	*p*-Value	Significance
*trans*-splicing		
scrASO vs. ASO7	<0.0001	***
scrASO vs. ASO9	<0.0001	***
*cis*-splicing exon 5/6-6/7		
scrASO vs. ASO7	0.0011	**
scrASO vs. ASO9	0.0011	**
*cis*-splicing exon 6/7-GFP		
scrASO vs. ASO7	0.0023	**
scrASO vs. ASO9	0.2506	ns

Significance values are given in asterisks (ns = not significant; ** *p*-value ≤ 0.01; *** *p*-value ≤ 0.001).

## Supplementary Materials

Supplementary materials can be found at http://www.mdpi.com/1422-0067/19/3/762/s1.

## Abbreviations

ASO	antisense oligonucleotide
asRNA	antisense RNA
BD	binding domain
EB	epidermolysis bullosa
EBS	epidermolysis bullosa simplex
DEB	dystrophic epidermolysis bullosa
*KRT14*-scMG	*KRT14* screening minigene
*KRT14*-scRTM	*KRT14* screening RTM
RDEB	recessive dystrophic epidermolysis bullosa
RTM	RNA *trans*-splicing molecule
SMaRT	Spliceosome mediated RNA *trans*-splicing
