# Supplementary material for: RNA Trans-Splicing Modulation via Antisense Molecule Interference
Source: Int J Mol Sci. 2018 Mar 7;19(3):762. doi: 10.3390/ijms19030762 (PMC5877623; doi:10.3390/ijms19030762)
Supplement: Supplementary file 1 [file ijms-19-00762-s001.pdf]

Supplementary Materials:

Figure S1: Prediction of splicing regulators within targeting regions of ASO1-9

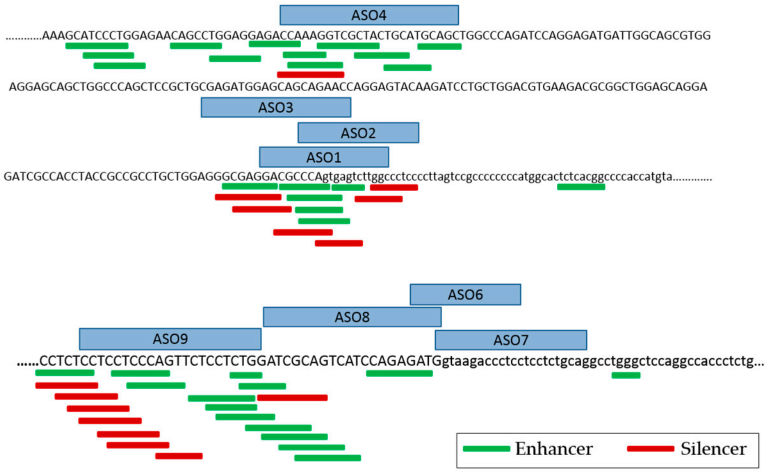

Designation	Sequence	Regulator
SF2/ASF(IgM) motif	CCGCCCAG, GCGAGGA,CCCAGTG	Enhancer
SC35 motif	GGCCCAGTG,CATCCCTG, GACCAAG, GGTCGCTA, GGTCGCTA, CGCTACTG	Enhancer
SF2/ASF(IgM)	CCCCAGTG, CCCTGGA, CAGCCTG, TGGAGGA, TGGAGGA, GAGACCA, CAAAGGT	Enhancer
PESE-Octamers	ACCCAGTGA, ACCAGTGA	Enhancer
SRp55	TTGAGTC, AGCATC, TGCAGC, TGCATG	Enhancer
ESS motif#3	AACGCCCAG, TGGCCCTC,ACGCCCAG	Silencer
ESS motif#2	AAGTGAGTC, TCTTGGCC, GGCGAGGA,CGAGGACG	Silencer
SRp40 motif	TCCCTGG, CCAAAG, CCAAAGG, CTACTGC	Enhancer
ESS motif#1	GGCGAGG, CCAAAGGT	Silencer

Figure S2: Agarose gel electrophoresis

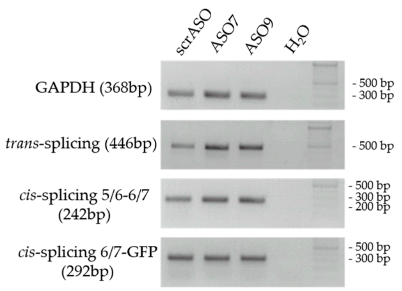

Figure S3: Prediction of splicing regulators within asRNA34 targeting region

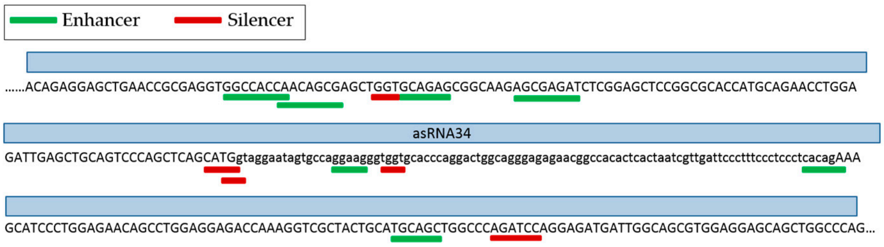

Designation	Sequence	Regulator
R0612 (ESE-SC35)	GGCCACCA	Enhancer
R0621 (ESE-SRp40)	TGCAGA, cacagA, TGCAGC	Enhancer
R0805	CATGg	Silencer
R0815	ggaag	Enhancer
R0836	AGATCC	Silencer
R0910	GAGCGAGA, GCGAGATG, GCAGGAGA	Enhancer
R0915	TGGT	Silencer
R0935	CAACAGCG	Enhancer
